# Inhaled bronchodilators and acute myocardial infarction: a nested case-control study

**DOI:** 10.1038/s41598-017-17890-1

**Published:** 2017-12-20

**Authors:** Chang-Hoon Lee, Seongmi Choi, Eun Jin Jang, Han-Mo Yang, Ho Il Yoon, Yun Jung Kim, Jimin Kim, Jae-Joon Yim, Deog Kyeom Kim

**Affiliations:** 1 National Evidence-based Healthcare Collaborating Agency, Namsan Square (Kukdong B/D) 173 Toegye-Ro, Jung-Gu, Seoul 04554 Republic of Korea; 2 Division of Pulmonary and Critical Care Medicine, Department of Internal Medicine, Seoul National University College of Medicine, Seoul National University Hospital, 101 Daehak-Ro, Jongno-Gu, Seoul 03080 Republic of Korea; 3 Department of Statistics, College of Natural Sciences, Kyungpook National University, 80 Daehakro, Buk-Gu, Daegu 41566 Republic of Korea; 4 Department of Information Statistics, Colloge of Natural Science, Andong National University, 1375 Gyeongdong-Ro, Andong, 36729 Republic of Korea; 5 Division of Cardiology, Department of Internal Medicine, College of Medicine, Seoul National University Hospital, 101 Daehak-Ro, Jongno-Gu, Seoul 03080 Republic of Korea; 6 Division of Pulmonary and Critical Care Medicine, Department of Internal Medicine, Seoul National University College of Medicine, Seoul National University Bundang Hospital, 82 Gumi-Ro, Bundang-Gu, Seongnam-Si, Gyeonggi-Do 13620 Republic of Korea; 7 Department of Health Policy and Hospital Management, Graduate School of Public Health, Korea University, Seongbuk-Gu, Seoul 02841 Republic of Korea; 8 Division of Pulmonary and Critical Care Medicine, Department of Internal Medicine, Seoul National University College of Medicine, Seoul Metropolitan Government-Seoul National University Boramae Medical Center, 20 Boramae-Ro 5-Gil, Dongjak-Gu, Seoul 07061 Republic of Korea

## Abstract

We investigated the association between the use of inhaled bronchodilators and the risk of AMI. A nested case-control study using the nationwide insurance claims database was conducted. Overall, 11,054 AMI cases and 47,815 matched (up to 1:5) controls were identified from 1,036,119 subjects without acute major cardiovascular events in the past year. Long-acting and short-acting β-agonists (LABAs and SABAs) were associated with increase in the risk of AMI, although an inhaled corticosteroid combined with a long-acting β-agonist was not. Long-acting muscarinic antagonists (LAMAs) in a dry powder inhaler (DPI) were significantly associated with reduced risk of AMI, while LAMAs in a soft mist inhaler (SMI) didn’t decrease the risk of it. In hypertensive or diabetic patients, LAMAs in a DPI were associated with reduced risk of AMI, but LABAs were associated with increased risk. Among the β-blocker users, the reduction of AMI risk by LAMAs was the most significant. In conclusions, inhaled β-agonists were associated with increase in the risk of AMI, while LABAs accompanied by ICSs were not associated with increase in the risk of AMI. LAMAs in a DPI use were associated with lower risk of AMI.

## Introduction

The efficacy of inhaled bronchodilator therapy has been proven in patients with airway diseases such as chronic obstructive pulmonary disease (COPD) and asthma^1,2^. Although inhaled therapy has advantages, such as rapid onset and fewer side effects compared with systemic administration, there have been concerns about the possibility of systemic adverse effects, including cardiovascular adverse events, because the drugs could be absorbed systemically after inhalation^3^. Of the possible cardiovascular adverse events, acute myocardial infarction (AMI) has been regarded as one of the most important issues concerning drug safety.

However, there are debates about the link between the use of inhaled bronchodilators, including inhaled β2–agonists^4–9^ and anti-cholinergics^10–14^, and the development of AMI. In addition, there are also debates regarding the impact that the drug-delivery device has on the patient outcome^13,15^. Although several randomized controlled trials (RCTs) yielded important information concerning drug safety, there are a limited number of RCTs with which to verify the differences in the development of adverse events. These studies often lack external validation^16–18^ and statistical power.

We investigated whether the use of inhaled bronchodilators affects the risk of AMI by using the nationwide database in South Korea.

## Results

**Figure 1:**
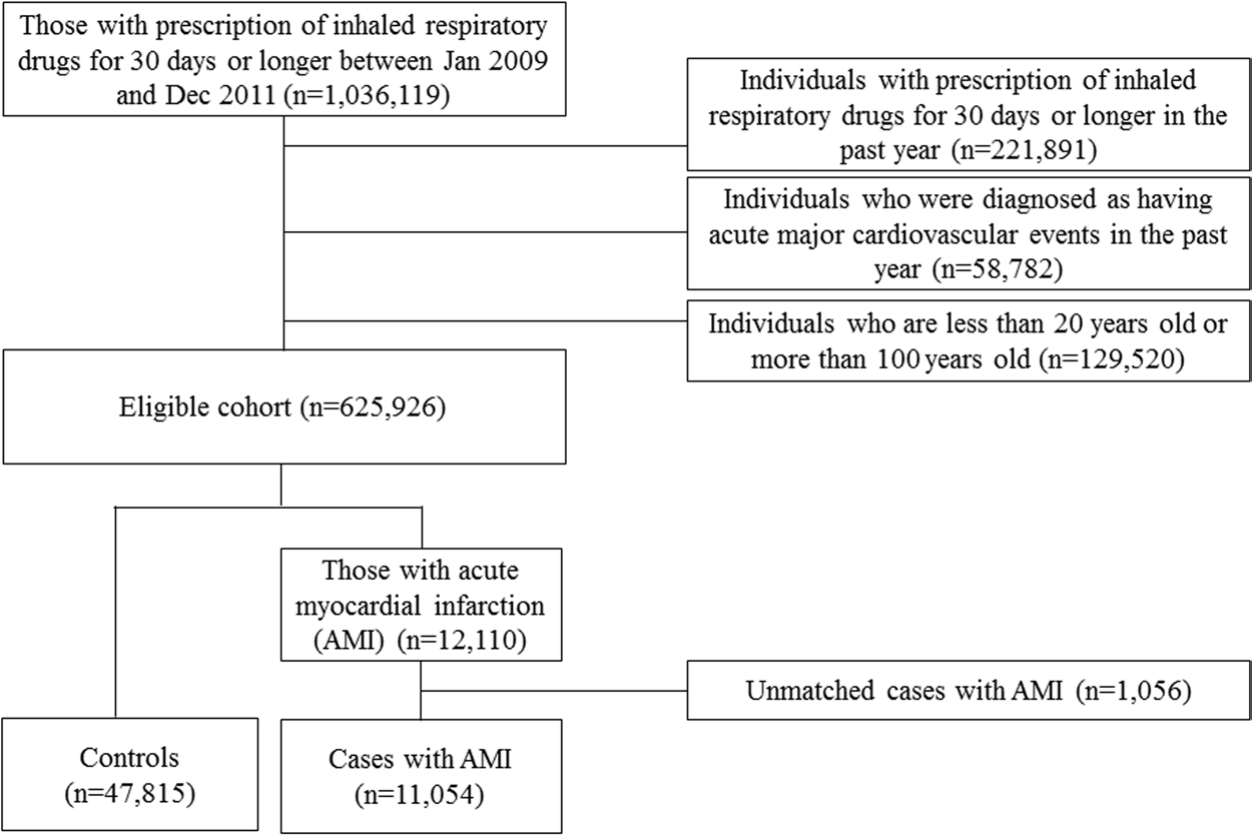
Flowchart for selecting cases and controls.

In total, 1,036,119 individuals with prescriptions of inhaled respiratory drugs for 30 days or longer between January 1, 2009, and December 31, 2011, were identified from the database. Among them, 221,891 individuals had previous prescriptions for inhaled respiratory drugs for 30 days or longer during the year prior to the initiation of the current therapy of inhaled respiratory medication; 58,782 individuals were diagnosed as having an AMI during the 1-year period before the index date; and 129,520 individuals were <20 years old, >100 years old, or of unknown age; all of these groups were excluded. Finally, a cohort of 792,687 new users of inhaled respiratory drugs were identified. During the study period, 12,110 individuals in this cohort were diagnosed with AMI. After excluding 1,056 (8.7%) cases who did not have matched controls, 11,054 cases with AMI and 47,815 matched controls were included in the analysis (Fig. 1).

There were statistically significant differences because of the large sample size. However, the majority of covariates, including other chronic respiratory diseases, comorbid dyslipidemia and the concomitant use of ACEI/ARB, statin, thiazide and calcium channel blocker, were well balanced between the cases with AMI and the controls because of extensive matching (Table 1). We used four statistical models to evaluate the association between inhaled drugs and AMI. In all of the models, LABAs and SABAs were associated with increase in the risk of AMI even after adjustment for the covariates that showed statistically significant differences between cases and controls (LABA, model 1; aOR, 1.30; 95% CI, 1.05–1.62, model 2; aOR, 1.30; 95% CI, 1.05–1.62, model 3; aOR, 1.32; 95% CI, 1.07–1.63, model 4; aOR, 1.4; 95% CI, 1.12–1.76, SABA, model 1; aOR, 1.20; 95% CI, 1.10–1.32, model 2; aOR, 1.20; 95% CI, 1.10–1.32, model 3; aOR, 1.20; 95% CI, 1.10–1.32). ICSs or ICSs combined with LABA was not associated with increase in AMI risk. (ICS, model 1; aOR, 0.88; 95% CI, 0.72–1.07; model 2; aOR, 0.88; 95% CI, 0.72–1.07; model 3; aOR, 0.91; 95% CI, 0.76–1.09; model 4; aOR, 0.89; 95% CI, 0.73–1.09, ICSs with LABAs, model 1; aOR, 1.04; 95% CI, 0.97–1.11, model 2; aOR, 1.04; 95% CI, 0.97–1.11, model 3; aOR, 1.04; 95% CI, 0.97–1.11, model 4; aOR, 1.03; 95% CI, 0.95–1.11) LAMAs in a DPI were significantly associated with reduced risk of AMI (model 2, aOR, 0.91; 95% CI, 0.83–0.99), while LAMAs in a SMI were not. (model 2, aOR, 1.05; 95% CI, 0.71–1.55) (Table 2). We did not find statistically significant dose-responses in the associations between either LABAs or LAMAs and the risk of AMI.

**Table 1:** Baseline characteristics of AMI cases and controls.

	AMI (N = 11,054)		Control (N = 47,815)		*P*-value
	n	(%)	n	(%)	
Sex					
Men	5,928	(53.6%)	25,288	(52.9%)	Matched
Women	5,126	(46.4%)	22,527	(47.1%)	
Age^1)^					
Mean ± SD	67.8 ± 12.1		67.5 ± 11.6		Matched
20–49	940	(8.5%)	3,813	(8.0%)	
50–59	1,474	(13.3%)	6,288	(13.2%)	
60–69	2,971	(26.9%)	13,753	(28.8%)	
70–79	4,017	(36.3%)	18,314	(38.3%)	
≥80	1,652	(14.9%)	5,647	(11.8%)	
COPD	6,278	(56.8%)	27,278	(57.0%)	Matched
Other chronic respiratory diseases^2),3)^					
TB-lung(B90)	403	(3.6%)	1,486	(3.1%)	0.008
Bronchiectasis(J47)	697	(6.3%)	2,892	(6.0%)	
Asthma(J45–46)	7,584	(68.6%)	33,393	(69.8%)	
Others	2,370	(21.4%)	10,044	(21.0%)	
Comorbidities^2)^					
Hypertension	7,831	(70.8%)	34,195	(71.5%)	Matched
Diabetes mellitus	4,968	(44.9%)	21,480	(44.9%)	Matched
Dyslipidemia	2,573	(23.3%)	8,971	(18.8%)	<0.001
Ischemic heart diseases	4,318	(39.1%)	17,133	(35.8%)	Matched
Other heart diseases (rheumatic diseases, cardiomyopathies, arrhythmias, valvular diseases, pericardial diseases)	3,493	(31.6%)	12,885	(26.9%)	Matched
Chronic kidney disease or dialysis	1,174	(10.6%)	3,410	(7.1%)	<0.001
Current concomitant medication^4)^					
ACEI/ARB	1,616	(14.6%)	7,696	(16.1%)	0.000
β-blocker	821	(7.4%)	3,354	(7.0%)	0.128
Statin	933	(8.4%)	4,382	(9.2%)	0.017
Aspirin	1,159	(10.5%)	4,891	(10.2%)	0.425
Thiazide	883	(8.0%)	4,466	(9.3%)	<0.001
Calcium channel blocker	1,468	(13.3%)	7,216	(15.1%)	<0.001
Concomitant medication^5)^					
ACEI/ARB	3,567	(32.3%)	16,588	(34.7%)	<0.001
β-blocker	1,848	(16.7%)	7,891	(16.5%)	0.584
Statin	2,190	(19.8%)	10,178	(21.3%)	0.001
Aspirin	2,665	(24.1%)	11,449	(23.9%)	0.715
Thiazide	2,092	(18.9%)	9,904	(20.7%)	<0.001
Calcium channel blocker	3,394	(30.7%)	15,954	(33.4%)	<0.001
MPR of Concomitant medication^6)^					
ACEI/ARB					
Mean ± SD	0.2 ± 0.3		0.3 ± 0.4		<0.001
Median(Q1, Q3)	0(0, 0.52)		0(0, 0.61)		
0	6,890	(62.3%)	28,929	(60.5%)	<0.001
0 < ≤ 0.3	705	(6.4%)	2,654	(5.6%)	
0.3 < ≤ 0.7	1,715	(15.5%)	7,091	(14.8%)	
0.7 < ≤ 1	1,744	(15.8%)	9,141	(19.1%)	
β-blocker					
Mean ± SD	0.1 ± 0.3		0.1 ± 0.3		0.708
Median(Q1, Q3)	0(0, 0)		0(0, 0)		
0	8,590	(77.7%)	37,843	(79.1%)	<0.001
0 < ≤ 0.3	729	(6.6%)	2,458	(5.1%)	
0.3 < ≤ 0.7	929	(8.4%)	3,611	(7.6%)	
0.7 < ≤ 1	806	(7.3%)	3,903	(8.2%)	
Statin					
Mean ± SD	0.1 ± 0.3		0.2 ± 0.3		<0.001
Median(Q1, Q3)	0(0, 0)		0(0, 0)		
0	8,386	(75.9%)	35,935	(75.2%)	<0.001
0 < ≤ 0.3	514	(4.6%)	1,815	(3.8%)	
0.3 < ≤ 0.7	1,189	(10.8%)	4,873	(10.2%)	
0.7 < ≤ 1	965	(8.7%)	5,192	(10.9%)	
Aspirin					
Mean ± SD	0.2 ± 0.3		0.2 ± 0.3		0.374
Median(Q1, Q3)	0(0, 0.19)		0(0, 0.17)		
0	7,738	(70.0%)	34,351	(71.8%)	<0.001
0 < ≤ 0.3	759	(6.9%)	2,259	(4.7%)	
0.3 < ≤ 0.7	1,404	(12.7%)	5,301	(11.1%)	
0.7 < ≤ 1	1,153	(10.4%)	5,904	(12.3%)	
Thiazide					
Mean ± SD	0.1 ± 0.3		0.1 ± 0.3		<0.001
Median(Q1, Q3)	0(0, 0)		0(0, 0)		
0	8,547	(77.3%)	36,230	(75.8%)	<0.001
0 < ≤ 0.3	563	(5.1%)	2,163	(4.5%)	
0.3 < ≤ 0.7	1,021	(9.2%)	4,268	(8.9%)	
0.7 < ≤ 1	923	(8.3%)	5,154	(10.8%)	
Calcium channel blocker					
Mean ± SD	0.2 ± 0.3		0.2 ± 0.4		<0.001
Median(Q1, Q3)	0(0, 0.47)		0(0, 0.57)		
0	7,010	(63.4%)	29,105	(60.9%)	<0.001
0 < ≤ 0.3	778	(7.0%)	3,219	(6.7%)	
0.3 < ≤ 0.7	1,652	(14.9%)	6,893	(14.4%)	
0.7 < ≤ 1	1,614	(14.6%)	8,598	(18.0%)	
Health care utilization^7)^					
Number of hospitalization					
Mean ± SD	1.3 ± 2.3		1.3 ± 2.4		0.062
Median(Q1, Q3)	0(0, 2)		0(0, 2)		
0	5,903	(53.4%)	23,991	(50.2%)	<0.001
1	2,283	(20.7%)	10,485	(21.9%)	
≥2	2,868	(25.9%)	13,339	(27.9%)	
Number of outpatient visit					
Mean ± SD	44.3 ± 42.4		44.4 ± 38.7		0.791
Median(Q1, Q3)	33(18,56)		34(20,56)		
<15	2,029	(18.4%)	7,172	(15.0%)	<0.001
15–30	3,132	(28.3%)	14,028	(29.3%)	
31–50	2,708	(24.5%)	12,424	(26.0%)	
>50	3,185	(28.8%)	14,191	(29.7%)	
Number of ER visit					
Mean ± SD	0.7 ± 1.5		0.7 ± 1.7		0.061
Median(Q1, Q3)	0(0, 1)		0(0, 1)		
0	7,147	(64.7%)	31,702	(66.3%)	0.001
≥1	3,907	(35.3%)	16,113	(33.7%)	

*p-values were derived from independent t-test for continuous variables and χ2-test for categorical variables, respectively.

^1^Age at initiation date.

^2^During 1-year period before index date until index date.

^3^Respiratory disease priority: TB-lung > Bronchiectasis > Asthma > Others.

^4^14 days or longer within 30 days prior to index date.

^5^Either more than 30 days or more than twice on prescription within 90 days prior to index date.

^6^Medication possession ratio (MPR) within 90 days prior to index date.

^7^Within 1-year prior to index date.

**Table 2:** Risk of acute myocardial infarction (AMI) according to inhaled drug use.

	AMI (N = 11,054)		Control (N = 47,815)		Unadjusted		Adjusted ^1)^		Adjusted ^2)^	
	n	(%)	n	(%)	OR (95% CI)	P-value	OR (95% CI)	*P*-value	OR (95% CI)	*P*-value
**Model 1**										
ICS, LABA										
Neither ICS nor LABA	9,433	(85.3%)	40,738	(85.2%)	—	—	—	—	—	—
ICS without LABA	134	(1.2%)	665	(1.4%)	0.86 (0.71, 1.05)	0.132	0.87 (0.72, 1.06)	0.157	0.88 (0.72, 1.07)	0.187
ICS with LABA	1,377	(12.5%)	6,023	(12.6%)	1 (0.94, 1.07)	0.984	1.01 (0.94, 1.08)	0.808	1.04 (0.97, 1.11)	0.313
LABA	110	(1.0%)	389	(0.8%)	1.28 (1.03, 1.59)	0.027	1.29 (1.04, 1.6)	0.023	1.3 (1.05, 1.62)	0.018
LAMA	717	(6.5%)	3,443	(7.2%)	0.91 (0.83, 0.99)	0.036	0.91 (0.83, 0.99)	0.033	0.92 (0.84, 1.01)	0.067
SABA	761	(6.9%)	2,832	(5.9%)	1.17 (1.07, 1.28)	0.001	1.17 (1.07, 1.28)	<0.001	1.2 (1.1, 1.32)	<0.001
**Model 2**										
ICS, LABA										
Neither ICS nor LABA	9,433	(85.3%)	40,738	(85.2%)	—	—	—	—	—	—
ICS without LABA	134	(1.2%)	665	(1.4%)	0.86 (0.71, 1.05)	0.132	0.87 (0.72, 1.05)	0.157	0.88 (0.72, 1.07)	0.186
ICS with LABA	1,377	(12.5%)	6,023	(12.6%)	1 (0.94, 1.07)	0.984	1.01 (0.94, 1.08)	0.810	1.04 (0.97, 1.11)	0.313
LABA	110	(1.0%)	389	(0.8%)	1.28 (1.03, 1.59)	0.027	1.29 (1.04, 1.6)	0.023	1.3 (1.05, 1.62)	0.018
LAMA SMI	33	(0.3%)	138	(0.3%)	1.08 (0.73, 1.59)	0.706	1.04 (0.71, 1.54)	0.833	1.05 (0.71, 1.55)	0.816
LAMA DPI	685	(6.2%)	3,314	(6.9%)	0.9 (0.82, 0.99)	0.025	0.9 (0.82, 0.99)	0.024	0.91 (0.83, 0.99)	0.0498
SABA	761	(6.9%)	2,832	(5.9%)	1.17 (1.07, 1.28)	0.001	1.17 (1.07, 1.28)	<0.001	1.2 (1.1, 1.32)	<0.001
**Model 3**										
ICS	148	(1.3%)	714	(1.5%)	0.89 (0.74, 1.07)	0.213	0.90 (0.75, 1.08)	0.264	0.91 (0.76, 1.09)	0.303
LABA	118	(1.1%)	412	(0.9%)	1.30 (1.06, 1.61)	0.013	1.31 (1.06, 1.61)	0.012	1.32 (1.07, 1.63)	0.011
ICS/LABA	1,375	(12.4%)	6,022	(12.6%)	1.00 (0.93, 1.07)	0.969	1.01 (0.94, 1.08)	0.820	1.04 (0.97, 1.11)	0.321
LAMA	717	(6.5%)	3,443	(7.2%)	0.91 (0.83, 0.99)	0.036	0.91 (0.83, 0.99)	0.033	0.92 (0.84, 1.01)	0.066
SABA	761	(6.9%)	2,832	(5.9%)	1.17 (1.07, 1.28)	0.001	1.17 (1.07, 1.28)	<0.001	1.2 (1.1, 1.32)	<0.001
**Model 4**										
None or SABA only	8,995	(81.4%)	38,603	(80.7%)	—	—	—	—	—	—
ICS only	125	(1.1%)	607	(1.3%)	0.88 (0.72, 1.07)	0.190	0.88 (0.72, 1.08)	0.222	0.89 (0.73, 1.09)	0.262
LABA only	106	(1.0%)	346	(0.7%)	1.38 (1.1, 1.72)	0.005	1.39 (1.11, 1.73)	0.004	1.4 (1.12, 1.76)	0.003
LAMA only	453	(4.1%)	2,186	(4.6%)	0.91 (0.81, 1.01)	0.076	0.91 (0.81, 1.01)	0.085	0.92 (0.82, 1.03)	0.144
ICS + LABA	1,111	(10.1%)	4,816	(10.1%)	1 (0.93, 1.08)	0.957	1 (0.93, 1.08)	0.954	1.03 (0.95, 1.11)	0.510
ICS + LAMA	9	(0.1%)	58	(0.1%)	0.66 (0.33, 1.35)	0.258	0.66 (0.33, 1.35)	0.257	0.67 (0.33, 1.37)	0.271
LABA + LAMA	4	(0.0%)	43	(0.1%)	0.4 (0.14, 1.14)	0.085	0.4 (0.14, 1.13)	0.085	0.4 (0.14, 1.16)	0.092
ICS + LABA + LAMA	251	(2.3%)	1,156	(2.4%)	0.95 (0.82, 1.09)	0.459	0.94 (0.81, 1.09)	0.399	0.98 (0.84, 1.13)	0.750

^1^Adjusted by other inhaled medication.

^2^Adjusted by other inhaled medication, age, other chronic respiratory disease, chronic kidney disease or dialysis, dyslipidemia, number of hospitalization, number of outpatient visit, number of ER visit, concomitant medication of ACEI/ARB, beta-blocker, statin, aspirin, thiazide, CCB.

**Figure 2:**
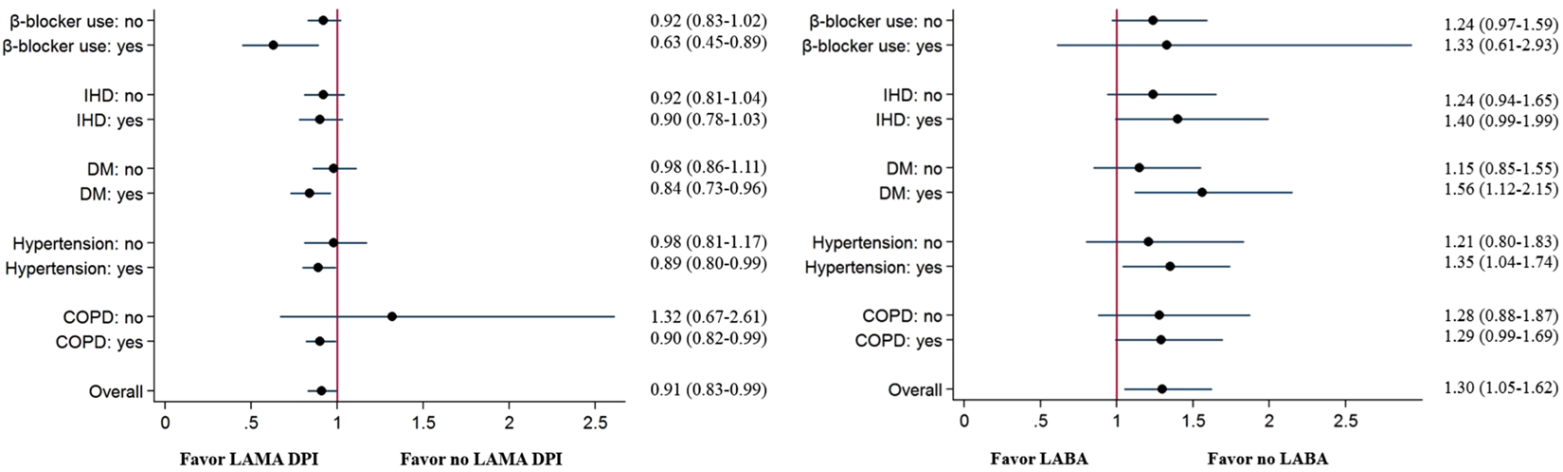
Subgroup analysis for the risks of LABA and LAMA for AMI. (**A**) LAMA. (**B**) LABA.

In the subgroup analyses, the AMI-reducing effects of LAMAs in a DPI were found in COPD patients (model 2, aOR, 0.90; 95% CI, 0.83–0.99), patients with hypertension (model 2, aOR, 0.89; 95% CI, 0.80–0.99), diabetes mellitus (model 2, aOR, 0.84; 95% CI, 0.73–0.96) and who used β-blocker (model 2, aOR, 0.63; 95% CI, 0.45–0.89) (Fig. 2(A)). On the contrary, LABAs were associated with increased risk of AMI among patients with hypertension (model 2, aOR, 1.35; 95% CI, 1.04–1.74) and those with diabetes mellitus (model 2, aOR, 1.56; 95% CI, 1.12–2.15) (Fig. 2(B)).

## Discussion

This nested case-control study showed that the use of inhaled LABAs but not ICSs with LABAs was associated with an increased risk of AMI, which contradicts the results of a post-hoc analysis of the TORCH trial^7^. Inhaled SABA use was also associated with increased risk of AMI in our study. In contrast, inhaled LAMAs in a DPI was significantly associated with reduced risk of AMI, which is in agreement with the results of the UPLIFT trial^11^.

The difference in the risk of AMI between inhaled β2-agonists and anti-cholinergics, which both increase the heart rates that might lead to ischemic events^19^, maybe because β2-agonists likely have a stronger effect on the cardiovascular system than cholinergic antagonism does. In fact, β-antagonists are considered important cardioprotective agents because of their anti-ischemic effects^20^. β2-adrenergic receptor stimulation not only increases the heart rate, which increases myocardial demand but also induces hypokalemia^21^ and causes direct myocardial injury or necrosis that could lead to ischemia^22,23^. β2-adrenoceptors are also present on numerous inflammatory cells, which could contribute to their potential for inducing adverse effects^24,25^. Our study also showed that LABAs combined with ICSs, potent anti-inflammatory drugs, did not increase the risk of AMI. In fact, there was a study reporting that low dose ICSs reduces AMI risk in patients with COPD^26^. The analysis of large RCTs showed there was a lower risk of cardiovascular adverse events in ICS-LABA combination group than there was in the LABA alone group^7,27^. This finding supports our results that the combined use of ICS/LABA could be a safer option than treatment with LABA alone.

Although, the provocative effects of anti-cholinergics on cardiovascular events might be weaker than those of β_2_–agonists, their vagolytic nature leads to arrhythmia^28^, and pro-inflammatory effects^10,29,30^ has been suggested as the mechanisms of higher cardiovascular events in anti-cholinergics users. Contrary to these hypotheses, our analysis revealed that inhaled LAMAs in a DPI (Handihaler) were associated with reduced risk of AMI in the total cohort and the COPD subgroup. This corresponds with the results of the UPLIFT trial^11^. However, inhaled LAMAs in an SMI were not associated with reduced risk of AMI, and the aOR was over 1.0, which is similar to the findings reported in several studies noting that LAMAs had a higher risk in an SMI than they did compared with LAMAs in a DPI^14,31^. The usual dosage (18 μg in LAMA DPI and 5 μg in LAMA SMI) might have been responsible for the different results. However, we did not find any dose-response relationship, and several studies showed similar safety profiles between these types of devices^12,32^. These effect of inhaled LABAs and LAMAs in a DPI on the risk of AMI was not observed in the patients without COPD. The protective effects of inhaled LAMAs were most predominant in β-blocker users, but the effect of LABAs on AMI did not significantly differ with the use of β-blockers.

Our study has advantages over previous studies including the large number of included RCTs. First, an RCT does not reflect the clinical situation. RCTs are designed to answer one question designated as the primary outcome, which usually has a narrow scope focused on the efficacy of the drug, and therefore, the RCT study design can be impractical. The strict inclusion and exclusion criteria in efficacy trials prevent the extrapolation of the study results to the general population, and the intensive follow-up schedule seldom occurs in usual clinical practice^16^. In addition, a higher dropout rate in the placebo group than that in the test group could lead to an underestimation of the incidence of adverse events in RCTs^17,18^. In our population-based, nested case-control study, the protective effects of LAMAs in a DPI on AMI were observed in the patients with hypertension or diabetes mellitus, whereas LABAs significantly increased the risk of AMI among these subgroups. This finding suggests that LAMAs rather than LABAs could be the preferred option in the selection of an inhaled bronchodilator for these AMI-susceptible patients. Second, RCTs were not designed to evaluate the significant differences in adverse events because these are not the primary outcomes; therefore, the statistical power could be insufficient and type I errors might occur. Our nested case-control study based on nationwide claim data included more than 600,000 individuals and detected more than 10,000 new AMI cases without specific exclusion criteria, which may reflect the reality of the clinical situation and could have sufficient statistical power. There could be concerns that many confounders might result in the misinterpretation of the results of a retrospective analysis. However, the distribution of confounding variables after our extensive matching in the analysis was well balanced in this study, as shown in Table 1. However, there were also numerous statistically significant differences in several covariates because of the large sample size.

Our study also has several limitations. First, we did not find statistically significant dose-response in the associations between either LABAs or LAMAs and the risk of AMI. Second, the LABAs were either salmeterol or formoterol, and all the LAMAs were tiotropium. The new LABAs including indacaterol, vilanterol and olodaterol, and new LAMAs including glycopyrronium, umeclidinium and acclidinium, were not used in South Korea until 2011. Third, our database did not include information about smoking, which is an important risk factors of both COPD and AMI. Fourth, we used dispensed prescriptions for inhaler use and did not directly confirm documented use. Fifth, although we observed the different effects on AMI between LAMA DPIs and LAMA SMI, the plausible underlying mechanisms could not be verified.

In conclusions, our population-based, nested case-control study revealed that inhaled β2–agonists alone were associated with increased risk of AMI, while LABAs in combination with ICSs were not associated with an increase in the risk of AMI. Inhalation of LAMAs using a DPI were associated with reduced risk of AMI. Finally, the results of our population-based, nested case-control study could facilitate the selection of appropriate inhaled drugs.

## Methods

### Source of data

We used the Health Insurance Review and Assessment Service (HIRA; Seoul, South Korea) database, which included 50.9 million South Koreans from the National Health Insurance (NHI) and National Medical Aid (NMA) databases. The HIRA database contains information on the demographics and all of the medical services rendered, along with the diagnostic codes (International Statistical Classification of Diseases and Related Health Problems, 10th edition code, ICD-10 code) and all of the medications prescribed. Values in key fields such as drug name, quantity, date dispensed, and duration are missing or out of range in <0.5% of the records. This study was approved by the ethics review committee of the National Evidence-based Healthcare Collaborating Agency, Seoul, Republic of Korea. Informed consent was waived because of the retrospective fashion by the ethics review committee. We followed the STROBE guideline for observational studies.

### Study design and study population

A nested case-control study was conducted based on the information from the HIRA database. The source population consisted of all of the individuals who were dispensed inhaled respiratory drugs for 30 days or longer between January 1, 2009 and December 31, 2011. The initiation date was defined as the date of the first use of the inhaled respiratory drugs in the hospital or at an outpatient visit. We excluded the following patients from this cohort: those who had prescriptions for inhaled respiratory drugs for 30 days or longer during the year prior to the initiation date; those who were diagnosed as having cardiovascular disease during the year prior to the initiation date; and those who were under 20 years of age or over 100 years of age. The detailed patient selection flow is presented in Fig. 1, and the final eligible cohort included 625,926 new users of inhaled respiratory drugs.

### Definition of cases and cardiovascular disease

Within the eligible cohort, we identified case individuals based on an ICD-10 diagnosis of AMI (I21-I24) that occurred after the initiation date of the inhaled respiratory drugs. The date of the first assignment of the AMI ICD-10 codes was called the index date.

### Definition of controls

We performed individual matching to select control patients for each case. The control patients were selected from the patients without ICD-10 codes for AMI. Each case was matched with up to five controls based on matching variables such as age (±5 years old), sex, initiation date of inhalers (±15 days), diagnosis of hypertension (ICD-10 code I10-I15), diabetes mellitus (DM; ICD-10 code E10-E14), COPD (ICD-10 code J41), ischemic heart disease (IHD; ICD-10 code I20, I25), diagnosis of other heart disease one year before the index date, or a Charson comorbidity index (CCI) of one year before the index date. Other heart disease was defined as rheumatic disease (ICD-10 code I00-I09) and cardiomyopathies, arrhythmias, valvular diseases, pericardial diseases (ICD-10 code I30-I52). The CCI variable was categorized into the following three groups: 0–1, 2–3, and ≥4. The index date for the controls was defined as the index date of the matched case.

### Exposure to inhaled medications

Inhaled drugs included ICSs (beclomethasone, budesonide, triamcinolone, ciclesonide, fluticasone, or flunisolide), a short-acting inhaled β2 agonist (SABA; salbutamol, fenoterol, procaterol, or terbutaline), a long-acting inhaled β2 agonist (LABA; salmeterol or formoterol), a short-acting inhaled muscarinic antagonist (SAMA; ipratropium), a long-acting inhaled muscarinic antagonist (LAMA; tiotropium), a combination of a SABA and SAMA (ipratropium and salbutamol), or a combination of a LABA and an ICS (budesonide/formoterol or fluticasone/salmeterol). Inhaler users were defined when they used inhaled drugs for 30 days or longer during one year, and respiratory drugs requiring a nebulizer were excluded in this study.

When we assessed the risk of AMI for each inhaler, the patient was defined as an inhaler user if the inhaler prescription was for 30 days or longer and was identified during the 90 days period before index date. If each inhaler prescription was for less than 30 days during the 90-day period before the index date, the patient was considered a non-user.

### Covariates

We considered the covariates for the AMI risk adjustment as the following: other chronic respiratory disease, comorbidities, health care utilization, and concomitant medications. The other chronic respiratory disease were classified as tuberculosis-lung (ICD-10 code B90), bronchiectasis (ICD-10 code J47), asthma (ICD-10 code J45–46), and others. Comorbidities included chronic kidney disease or dialysis (ICD-10 code N17-N19) and dyslipidemia (ICD-10 code E780, E789). We used health care utilization, such as number of hospitalizations (0, 1, ≥2), outpatient visits (<15, 15–30, 31–50, >50), and emergency room (ER) visits (0, ≥1), to adjust patient severity. Concomitant medications included angiotensin converting enzyme inhibitors (ACEI)/angiotensin receptor blockers (ARB), beta-blockers, statins, aspirins, thiazides, and calcium-channel blockers (CCB).

### Statistical analysis

The baseline characteristics of the cases and controls were summarized by descriptive statistics, such as proportion, mean, standard deviation (SD), median, first quartile (Q1), and third quartile (Q3). We also summarized the continuous variables into the appropriated categorical variables based on their distributions. Statistical significances were derived from an independent t-test for continuous variables and a χ2-test for categorical variables.

The association between the use of inhaled respiratory medication and AMI was investigated by conditional logistic regression analysis. We adjusted for the following covariates: age, other chronic respiratory disease, chronic kidney disease or dialysis, dyslipidemia, use of concomitant medications, number of hospitalization, outpatients visit, and ER visits. Unadjusted odds ratios (ORs) and adjusted odds ratios (aORs) are presented with a 95% confidence interval (CI).

Subgroup analyses for LAMAs and LABAs were conducted according to beta-blocker use, IHD, DM, hypertension, and COPD.

A p-value less than 0.05 was regarded as statistically significance, and all of the statistical analyses were performed using SAS V.9.2 (SAS Institute, Cary, NC, USA).
